# Evaluation of maternal serum protein biomarkers in the prenatal evaluation of placenta accreta spectrum: A systematic scoping review

**DOI:** 10.1111/aogs.14918

**Published:** 2024-07-14

**Authors:** Matthew Givens, Ivaila Valcheva, Brett D. Einerson, Ewelina Rogozińska, Eric Jauniaux

**Affiliations:** ^1^ Department of Obstetrics and Gynecology (Drs Givens and Einerson) University of Utah Health (UUH) Salt Lake City Utah USA; ^2^ EGA Institute for Women's Health, Faculty of Population Health Sciences University College London London UK; ^3^ The EVIdencE Synthesis and Methodology Group for Women's Health Research (EVIE) Institute of Clinical Trials & Methodology, University College London London UK

**Keywords:** maternal serum, placenta accreta, placenta accreta spectrum, placenta previa accreta, protein biomarkers, screening

## Abstract

**Introduction:**

Placenta accreta spectrum (PAS) is an increasingly commonly reported condition due to the continuous increase in the rate of cesarean deliveries (CD) worldwide; however, the prenatal screening for pregnant patients at risk of PAS at birth remains limited, in particular when imaging expertise is not available.

**Material and Methods:**

Two major electronic databases (MEDLINE and Embase) were searched electronically for articles published in English between October 1992 and January 2023 using combinations of the relevant medical subject heading terms and keywords. Two independent reviewers selected observational studies that provided data on one or more measurement of maternal blood‐specific biomarker(s) during pregnancies with PAS at birth. PRISMA Extension for Scoping Review (PRISMA‐ScR) was used to extract data and report results.

**Results:**

Of the 441 reviewed articles, 29 met the inclusion criteria reporting on 34 different biomarkers. 14 studies were retrospective and 15 prospective overall including 18 251 participants. Six studies had a cohort design and the remaining a case–control design. Wide clinical heterogeneity was found in the included studies. In eight studies, the samples were obtained in the first trimester; in five, the samples were collected on hospital admission for delivery; and in the rest, the samples were collected during the second and/or third trimester.

**Conclusions:**

Measurements of serum biomarkers, some of which have been or are still used in screening for other pregnancy complications, could contribute to the prenatal evaluation of patients at risk of PAS at delivery; however, important evidence gaps were identified for suitable cutoffs for most biomarkers, variability of gestational age at sampling and the potential overlap of the marker values with other placental‐related complications of pregnancy.

## Graphical abstract

Measurements of serum protein biomarkers which have been or are still used in screening for fetal aneuploidy, fetal neuro‐tubal defects, fetal growth restriction, and preeclampsia may contribute to prenatal evaluation of patients at risk of placenta accreta spectrum at delivery but should be evaluated prospectively in case‐control studies matched for gestational age at sampling.

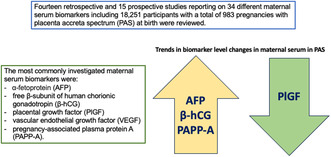

**Abbreviations**
AFP: alpha‐fetoprotein
CD: cesarean delivery
PAPP‐A: pregnancy‐associated plasma protein
PAS: placenta accreta spectrum
PIGF: placental growth factor
VEGF: vascular endothelial growth factor
β‐hCG: free β‐subunit of human chorionic gonadotropin

**Key message**

Serum protein biomarkers used in screening for fetal anomalies and preeclampsia may contribute to prenatal evaluation of patients at risk of placenta accreta spectrum at delivery but evidence gaps were found for suitable cutoffs and gestational age at sampling.

## 1 INTRODUCTION

Placenta accreta spectrum (PAS) is the consequence of the development of the gestational sac in a uterine scar area, mainly in a lower uterine segment (LUS) cesarean section scar.^1^, ^2^ After a cesarean delivery (CD), up to 70% of patients develop a cesarean scar defect (CSD) and are at risk of a cesarean scar ectopic pregnancy (CSEP) in subsequent pregnancies.^3^ More than half of ongoing first trimester CSEP are diagnosed with PAS at birth and the risk of CSD, CSEP, and PAS^4^, ^5^ with the number of prior CDs, indicating a common etiopathology.^2^

Pregnant patients with a history of multiple CDs, presenting with a low‐lying/placenta previa are at the highest risk of accreta placentation.^6^, ^7^, ^8^ As pregnancy advances, high‐velocity blood flow enters the intervillous space, distorting its anatomy.^2^, ^9^ This is associated with thick fibrinoid deposition at the utero‐placental interface, independently of deeply implanted villous tissue in 70% of the samples from the accreta areas.^10^ These changes are associated with distortion of the “Nitabuch's membrane” and might contribute to the loss of parts of the physiological site of detachment of the placenta from the uterine wall in PAS. When unsuspected at delivery, attempts to manually remove accreta placental tissue typically provoke rapid massive obstetric hemorrhage.^11^ The risk is particularly high when there is also extensive remodeling of the LUS after multiple CDs.^12^, ^13^ These cases often require complex surgical management by a specialised multidisciplinary team (MDT).^14^, ^15^

Prenatal identification of patients with a high probability of PAS at birth has been shown to decrease maternal morbidity.^16^, ^17^ However, if awareness of PAS has increased over the last decade, training programs for ultrasound operators and standardized screening protocols have not yet become widely available.^18^ Zelop et al. were the first to publish on the association between a maternal serum (MS) protein, elevated alpha‐fetoprotein (AFP), and PAS at birth.^19^ Several other maternal serum biomarkers have been investigated for their role in the prenatal diagnosis of PAS including markers of aneuploidy, cell‐free placental mDNA, or mRNA and factors associated with abnormal extravillous trophoblastic (EVT) migration.^20^, ^21^, ^22^ However, to date, there is no accurate serum marker for the antenatal diagnosis of PAS and the screening of patients at risk relies on obstetric historical factors and ultrasound imaging. The aim of this study was to summarize the literature on the use of maternal serum biomarkers in pregnancies complicated by PAS confirmed clinically or pathologically at birth and to identify protein biomarkers that may be useful in the prenatal screening of patients at high risk of PAS.

## 2 MATERIAL AND METHODS

### 2.1 Eligibility criteria, information sources, and search strategy

The review was guided by prospectively developed protocol and registered with PROSPERO (CRD42022332486). The data are reported in accordance with the Preferred Reporting Items for Systematic Reviews and Meta‐Analyses extension for Scoping Review (PRISMA‐ScR).^22^ We searched in MEDLINE (via PubMed) and Embase to identify studies examining an association between maternal biomarker and PAS published between October 1, 1992 (first known study on this topic by Zelop et al.)^19^ and January 15, 2023. We used the following medical subject headings (MeSH) terms: “placenta accreta” OR “placenta increta” OR “placenta percreta” OR “abnormally invasive placenta” OR “morbidly adherent placenta” OR “placenta previa accreta” AND “maternal blood” OR “maternal serum” OR “protein biomarkers”. We limited our search to articles published in English. The systematic search was supplemented with a hand search in Google Scholar and other sources including abstracts and proceedings of conferences and meetings as well as the reference lists of reviews and editorials.

### 2.2 Article selection

Two independent investigators (MG and IV) selected the studies in two stages: first, the abstracts of all potentially relevant articles were individually examined for suitability; secondly the remainder were obtained in full text and independently assessed for inclusion and inclusion criteria. Disagreements between the two original reviewers were resolved by discussion with the third investigator (EJ).

The studies had to meet the following criteria in order to be included in the review: (1) observational study (cohort or case control) including 10 or more pregnant patients with pregnancies complicated by PAS who had blood collected at any stage during pregnancy to evaluate an association between a specific protein biomarker and PAS at birth; (2) study had to report outcomes including confirmation of the diagnosis of PAS using delivery clinical criteria (abnormal attachment of part of the placenta to the uterine wall) and/or histopathology (absence of decidua with direct attachment of the villous tissue to the uterine wall). We excluded duplicates, case reports, small case series of <10 cases, letters, reviews, articles published before 1992, cohort studies of first trimester cesarean scar pregnancies diagnosed and terminated before 11 weeks of gestation (without possible confirmation of the diagnosis of PAS at birth) and reporting on cell‐free placental mRNA. All article retrieved were exported into Covidence (Covidence systematic review software. Veritas Health Innovation, Melbourne, Australia).

### 2.3 Data extraction

Two reviewers (MG and IV) independently assessed the content of the full text and resolved discrepancies through discussion with the other authors. We used a predesigned template to extraction data on study design, study type, sample size, recruitment setting, protein biomarker(s) investigated year of publication, country of origin, methodology, outcomes, and criteria used to confirm the diagnosis of PAS at birth.

### 2.4 Data synthesis

The evaluation of most biomarkers identified in the present review was limited due to low quality or availability of data for specific proteins and our study which was designed as a systematic review and meta‐analysis was re‐designed as a systematic scoping study. The extracted data for the biomarkers most commonly used in clinical practice and for which bioassays are available in most biology laboratories were evaluated for their suitability. The corresponding data were pooled using fixed or random effects model with inverse‐variance weighting as appropriate and analyzed using Review Manager version 5.1 (RevMan, Cochrane, UK). Due to low number of studies per analysis, we were not able to explore small study effect neither graphically (funnel plot) nor statistically.

## 3 RESULTS

**FIGURE 1:**
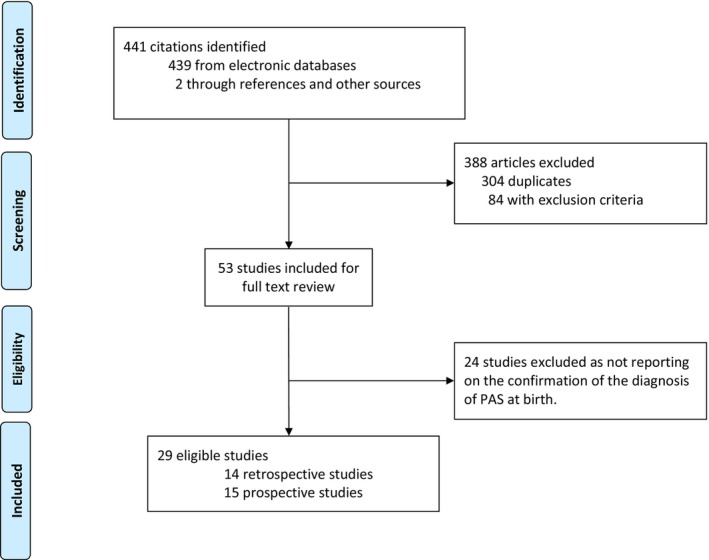
Flow diagram showing the selection of articles included in the review.

Out of 441 citations identified through literature search, 29 studies^19^, ^23^, ^24^, ^25^, ^26^, ^27^, ^28^, ^29^, ^30^, ^31^, ^32^, ^33^, ^34^, ^35^, ^36^, ^37^, ^38^, ^39^, ^40^, ^41^, ^42^, ^43^, ^44^, ^45^, ^46^, ^47^, ^48^, ^49^, ^50^ met our eligibility criteria for inclusion in this review and are reported in the PRISMA flow diagram (Figure 1).

### 3.1 Study characteristics

Characteristics of the included studies are presented in Table 1. There were 14 retrospective studies and 15 prospective studies, overall including 18 251 participants with a total of 983 pregnancies with PAS reported as clinically and/or pathologically confirmed at birth. Six studies had a cohort design, including one population cohort^24^ and five cohorts of patients presenting prenatally with a placenta previa.^25^, ^28^, ^30^, ^34^, ^36^ The other studies had a case–control design. All but two studies^42^, ^47^ were from a single institution.

**TABLE 1:** Characteristics of the included studies.

Author et al. (Year)	Country	Study period	Type of study	Selection criteria	Study design	Biomarker (GA)	Cases of PAS (%)
Zelop et al. (1992)^19^	USA	1986–1990	Retrospective/ single institution	Cases: Patients with placenta previa who underwent emergency PH for PAS Controls: Patients with placenta previa and no evidence of PAS at birth Exclusion: PH for other reasons than PAS	Case–control	MSAFP (second trimester)	11 PAS cases vs 14 previa non‐PAS
Kupferminc et al. (1993)^23^	USA	1985–1992	Retrospective/single institution	Cases: Patients with placenta previa who underwent emergency PH for PAS Controls: Patients who underwent emergency PH with no PAS at birth Exclusion: Cases where MSAFP not available	Case–control	MSAFP (second trimester)	20 PAS cases (including 14 previa) vs 24 controls (including 12 previa non‐PAS)
Hung et al. (1999)^24^	Taiwan	1994–1997	Retrospective/single institution	Patient with PAS and without PAS at birth Exclusion: Multiple gestations and Pregnancies complicated by diabetes and fetal malformations/Aneuploidy	Cohort of 9349 births	MSAFP Free β‐hCG (14–22 wks)	28 (0.3%) PAS cases (including 7 previa) and 9321 (including 68 previa non‐PAS)
Butler et al. (2001)^25^	USA	1990–1999	Retrospective/single institution	Patients with placenta previa on TAS Exclusion: Multiple gestations and intrauterine fetal death	Cohort of 107 births	MSAFP (15–20 wks)	15 (14%) PAS cases and 92 previa non‐PAS
Wehrum et al. (2011)^26^	USA	2005–2010	Prospective/single institution	Cases: Consecutive patients with complete placenta previa presenting with vaginal bleeding in the third trimester including 13 PAS confirmed at birth Controls: Patients with uncomplicated pregnancies matched by GA Exclusion: cerclage, placental abruption, history of transfusion or steroid treatment prior to enrolment, fetal heart rate abnormalities at enrolment, and maternal medical complications	Case–control	VEGF PlGF sFlt (28–34 wks)	13 PAS cases vs 45 normal controls
Dreux et al. (2012)^27^	France	2000–2009	Retrospective/single institution	Cases: Patients diagnosed with PAS at delivery Controls: Patients randomly chosen from a serum screening database matched by maternal age Exclusion: Multiple gestations	Case–control	MSAFP Intact hCG (second trimester)	69 PAS cases vs 552 controls (Placental location not described)
Desai et al. (2014)^28^	USA	2005–2011	Retrospective/single institution	Patients with placenta previa on US with PAS or without PAS at birth Exclusion: Fetal malformations/Aneuploidy	Cohort of 82 births	PAPP‐A Free β‐hCG (1st Trimester)	16 (19.5%) PAS cases and 66 previa non‐PAS
Duzyj et al. (2015)^29^	USA	2005–2012	Retrospective/single institution	Cases: Patients with placenta previa on PAS and US at birth Control: Patients with placenta previa without PAS at birth	Case–control	Soluble E‐CAD (25–35 wks)	23 PAS cases vs 21 previa non‐PAS
Thompson et al. (2015)^30^	UK	2005–2013	Retrospective/single institution	Patients with placenta previa on US with or without PAS at birth Exclusion: Fetal aneuploidy and other placental pathology	Cohort of 172 births	PAPP‐A Free β‐hCG (first trimester)	17 (9.9%) PAS cases and 155 previa non‐PAS
Biberoglu et al. (2016)^31^	Turkey	2014–2015	Prospective/single institution	Cases: Patients with complete placenta previa presenting with vaginal bleeding in the third trimester with PAS at birth Controls: Patients with placenta previa without PAS at birth and patients with normal placentation Exclusion: Multiple gestations, patients with systemic disorders, on medication and obese	Case–control	VEGF PlGF sFlt (third trimester)	35 PAS cases vs 33 previa non‐PAS vs 30 normal controls
Ersoy et al. (2016)^32^	Turkey	2014–2015	Prospective/single institution	Cases: Patients with placenta previa on US and PAS at birth Controls: Patients with placenta previa without PAS at birth and patients with normal placentation matched for maternal age and BMI Exclusion: Multiple gestations, patients with systemic disorders, premature rupture of membranes, smokers, and drug addiction	Case–control	Troponin ProBNP CK CK‐MB (at delivery)	14 PAS cases vs 40 previa non‐PAS vs 46 normal controls
Oztas et al. (2016)^33^	Turkey	2013–2014	Prospective/single institution	Cases: Patients with placenta previa on US and PAS at birth Controls: Patients with placenta previa without PAS at birth and patients with normal placentation matched for maternal age and BMI Exclusion: Multiple gestations, patients with systemic disorders, on medication and fetal malformations/Aneuploidy	Case–control	TRAIL‐R2 (at delivery)	27 PAS cases vs 26 previa non‐PAS vs 29 normal controls
Oztas et al. (2016)^34^	Turkey	2009–2015	Retrospective/single institution	Patients with placenta previa with or without PAS at delivery Exclusion: Multiple gestations, patients with systemic disorders, on medication and fetal malformations/ Aneuploidy	Cohort of 316 births	MSAFP PAPP‐A Free β‐hCG uE3 hCG (1st or 2nd trimester)	112 (35.4%) PAS cases including 61 managed conservatively and 51 requiring PH and 204 previa non‐PAS
Einerson et al. (2017)^35^	USA	N/A	Prospective/single institution	Cases: Consecutive patients with PAS confirmed at birth Controls: Patients with uncomplicated pregnancies matched for GA and risk factors for PAS Exclusion: Not provided	Case–control	hCG‐H (2nd and 3rd trimester)	30 PAS cases vs 30 controls (placental location not described)
Buke et (2018)^36^	Turkey	2011–2014	Retrospective/single institution	Patients with placenta previa with or without PAS at birth Exclusion: Not provided	Cohort of 88 births	PAPP‐A Free β‐hCG (1st trimester)	19 PAS (21.6%) and 69 previa non‐PAS
Uyanikoglu et al. (2018)^37^	Turkey	2015–2017	Prospective/single institution	Cases: Patients with placenta percreta at birth Controls: Patients with uncomplicated pregnancies matched for GA Exclusion: Multiple gestations, patients with systemic disorders, preeclampsia and FGR	Case–control	Native thiol Total thiol Disulfide IMA (at delivery)	38 PAS cases vs 40 controls (placental location not described)
Uyanikoglu et al. (2018)^38^	Turkey	2015–2016	Prospective/single institution	Cases: Patients with placenta percreta at birth Controls: Patients with uncomplicated pregnancies Exclusion: Multiple gestations, patients with systemic disorders, preeclampsia, and FGR	Case–control	VEGF PlGF sFlt‐1 (at delivery)	22 PAS cases vs 22 controls (placental location not described)
Berezowsky et al. (2019)^39^	Israel	2007–2014	Retrospective/single institution	Cases: Patients with placenta previa with or without PAS at birth Controls: Patients with normal placentation Exclusion: Multiple gestations	Case–control	MSAFP hCG uE3 (2nd trimester)	64 PAS cases vs 66 previa non‐PAS vs 17 previa PAS vs 153 normal controls
Penzhoyan et al. (2019)^40^	Russia	2015–2018	Retrospective/single institution	Cases: Patients with placenta previa and PAS at birth Controls: Patients with or without placenta previa and without PAS at birth Exclusion: Multiple gestations and fetal malformations/Aneuploidy	Case–control	PAPP‐A Free β‐hCG (1st trimester)	25 PAS cases vs 39 controls (including 23 previa non‐PAS)
Al Khan et al. (2020)^41^	USA	N/A	Prospective/single institution	Cases: Patients with PAS at birth Controls: Patients with placenta previa without PAS at birth and patients with normal placentation Exclusion: Not provided	Case–control	hCG‐H Decorin IL‐8 (≥ 25 weeks)	56 PAS cases including 42 previa–PAS vs 18 previa non‐PAS vs 14 normal controls
Shainker et al. (2020)^42^	Israel, USA	2017–2019	Prospective/multicentric	Cases: Patients with PAS at birth Controls: Patients with placenta previa and patients with normal placentation Exclusion: Not provided	Case–control	Antithrombin III PAI‐1 sTie2 sVEGFR‐2 (3rd trimester)	16 PAS cases vs 10 controls including 4 with normal placentation, 5 with placenta previa, and 1 unknown
Wang et al. (2020)^43^	China	2017–2019	Retrospective/single Institution	Cases: Patients with placenta previa and PAS at birth Controls: Patients with placenta previa without PAS at birth and patients with normal placentation matched for maternal age and BMI Exclusion: Multiple gestations, patients with systemic disorders, pregnancy complications, and stillbirth	Case–control	PlGF (1st trimester)	35 PAS cases vs 30 previa non‐PAS vs 112 normal controls
Faraji et al. (2021)^44^	Iran	2017–2019	Prospective/single institution	Cases: Patients with PAS at birth Controls: Patients with no PAS at birth Exclusion: Multiple gestations, patients with systemic disorders, pregnancy complications, and FGR	Case–control	PlGF VEGF (3rd trimester)	45 PAS cases vs 45 controls (placental location not described)
Ozler et al. (2021)^45^	Turkey	N/A	Prospective/single institution	Cases: Patients with placenta previa and PAS at birth Controls: Patients with placenta previa without PAS at birth and patients with normal placentation matched for maternal age and BMI Exclusion: Multiple gestations, patients with systemic disorders, pregnancy complications, and smokers	Case–control	IL‐33 IL‐6 CRP (>26 wks)	27 PAS cases vs 30 previa non‐PAS vs 30 normal controls
Sahin et al. (2021)^46^	Turkey	2019–2020	Prospective/single institution	Cases: Patients with PAS at birth Controls: Patients with uncomplicated pregnancies Exclusion: Preeclampsia, FGR and use of tocolytic agent	Case–Control	PP‐13 (3rd trimester)	41 PAS cases vs 32 controls (placental location not described)
Schwickert et al. (2021)^47^	Germany, Belgium	N/A	Prospective/multicentric	Cases: Patients with PAS at birth Controls: Patients with uncomplicated pregnancies matched for GA Exclusion: Multiple gestations	Case–control	NT‐proBNP VEGF (at delivery)	44 PAS cases vs 55 controls (including 5 with placenta previa)
Wang et al. (2021)^48^	China	2017–2019	Retrospective/single institution	Cases: Patients with placenta previa and PAS at birth Controls: Patients with normal placentation and patients with placenta previa non‐PAS matched for maternal age and BMI Exclusion: Multiple gestations, patients with systemic disorders, and stillbirth	Case–control	PAPP‐A (1st trimester)	35 PAS cases vs 30 previa non‐PAS vs 112 normal controls
Wang et al. (2021)^49^	China	2017–2020	Prospective/single institution	Cases: Patients with placenta previa and PAS at birth and patients with placenta previa non‐PAS Controls: Patients with normal placentation matched for smoking, GA, maternal age, and BMI Exclusion: Multiple gestations, patients with systemic disorders, and preeclampsia	Case–control	VEGF sFLT‐1 (not provided)	56 PAS cases and 84 previa non‐PAS vs 46 normal controls
Ozler al (2022)^50^	Turkey	2018	Prospective/single institution	Cases: Patients with placenta previa and PAS at birth Controls: Patients with placenta previa without PAS at birth and patients with normal placentation matched for maternal age and BMI Exclusion: Multiple gestations, patients with systemic disorders, obesity, premature rupture of membranes, and preeclampsia	Case–control	TSH TgAB T3 T4 TPOAb (3rd trimester)	30 PAS cases vs 28 previa non‐PAS vs 30 normal controls

*Note*: Normal controls refers to pregnancies with normal placentation (upper segment).

Abbreviations: AFP, alpha‐fetoprotein; ARES, antioxidant response elements; BMI, body mass index; β‐hCG, β‐subunit of human chorionic gonadotropin; CD, cesarean delivery; CK, creatine kinase; CK‐MB, cardiac form of CK; CRP, C‐reactive protein; E‐CAD, E‐cadherin; FGR, fetal growth restriction; hCG‐H, hyperglycosylated human chorionic gonadotropin; GA, gestational age; HP, histopathology; IL, interleukin; IMA, ischemia‐modified albumin; N/A, not available; NT‐proBNP, N‐terminal prohormone of brain natriuretic peptide; MS, maternal serum; OSI, oxidative stress index; PAS, placenta accreta spectrum; PAI‐1, plasminogen activator inhibitor‐1; PAPP‐A, pregnancy‐associated plasma protein A; PH, peripartum hysterectomy; PlGF, placenta growth factor; PP‐13, placental protein 13; ProBNP, pro‐brain natriuretic peptide; sFLT‐1, soluble fms‐like tyrosine kinase; sTie2, soluble Tie2; sVEGFR‐2, soluble VEGF receptor 2; T3, triiodothyronine; T4, thyroxine; TAS, total antioxidant status; TAS, transabdominal sonography; TgAB, thyroglobulin antibodies; TOS, total oxygen species; TPOAb, thyroid peroxidase antibodies; TRAIL‐R2, TNF‐related apoptosis‐inducing ligand receptor‐2; TSH, thyroid stimulating hormone; uE3, estriol; US, ultrasound; VEGF, vascular endothelial growth factor; Wks, weeks.

All patients included in the 27 studies reviewed had singleton pregnancies. Exclusion criteria were not provided in four studies.^35^, ^36^, ^41^, ^42^ 17 case–control studies compared cases presenting with a placenta previa accreta confirmed at birth with cases with a placenta previa but no evidence of PAS at birth.^19^, ^26^, ^29^, ^31^, ^32^, ^33^, ^34^, ^36^, ^39^, ^40^, ^41^, ^42^, ^43^, ^45^, ^48^, ^49^, ^50^ In nine of these case–control studies, the authors also compared the data of pregnancies with PAS confirmed at birth with those of uncomplicated pregnancies with normal placentation.^26^, ^31^, ^32^, ^39^, ^41^, ^43^, ^45^, ^48^, ^50^ In five, all case–control studies, the samples were collected on admission for delivery.^32^, ^33^, ^37^, ^38^, ^47^ In the other studies, the samples were collected during the second and/or third trimester and in one study^49^ there was no data on the gestational age at sampling.

We identified 34 different protein biomarkers tested in patients with PAS (Table 1). The most commonly investigated MS biomarkers were AFP,^19^, ^20^, ^21^, ^22^, ^23^, ^24^, ^25^, ^27^, ^34^, ^39^, ^51^ free β‐subunit of human chorionic gonadotropin (β‐hCG),^24^, ^28^, ^30^, ^34^, ^37^, ^40^ placental growth factor (PIGF),^26^, ^31^, ^38^, ^43^, ^44^, ^48^ vascular endothelial growth factor (VEGF),^26^, ^31^, ^38^, ^44^, ^49^ and pregnancy‐associated plasma protein A (PAPP‐A).^28^, ^30^, ^34^, ^36^, ^40^ In eight studies, the samples were obtained in the first trimester (11 weeks and 1 day–13 weeks and 6 days) of pregnancy as part of the screening for fetal aneuploidy^28^, ^30^, ^34^, ^36^, ^40^, ^48^ or for preeclampsia risk assessment.^43^, ^44^ In the other studies, the samples were obtained during the second trimester,^19^, ^23^, ^24^, ^27^, ^39^ during the third trimester,^26^, ^42^, ^44^, ^46^, ^50^ during the second and third trimester^29^, ^31^, ^35^, ^41^, ^45^ or at delivery.^32^, ^33^, ^37^, ^38^, ^47^ In one study, there was no description of the gestational age at sampling.^49^ 13 authors reported their measurements as multiple of the median (MoM)^19^, ^23^, ^24^, ^25^, ^27^, ^28^, ^30^, ^34^, ^36^, ^39^, ^40^, ^43^, ^48^ and the others as mean or median (Table S1).

The use of prenatal ultrasound imaging to evaluate the placental location was reported in 22 studies,^25^, ^26^, ^27^, ^28^, ^29^, ^30^, ^31^, ^32^, ^33^, ^34^, ^37^, ^38^, ^39^, ^40^, ^41^, ^43^, ^44^, ^45^, ^46^, ^47^, ^48^, ^49^, ^50^ including in seven, a description of the ultrasound signs associated with PAS at birth (Table 2).^26^, ^31^, ^37^, ^38^, ^45^, ^46^, ^47^ Five studies also reported on the use of magnetic resonance imaging (MRI)^30^, ^40^, ^41^, ^44^, ^49^ and three on the use of transvaginal sonography (TVS).^26^, ^31^, ^46^ One study used standardized ultrasound signs proposed by the European Working Group on abnormally invasive placenta.^52^ No study compared the change in biomarker levels with the imaging findings or if the combined use of biomarkers and imaging had an impact of the antenatal detection rate of patients at risk of PAS at birth.

**TABLE 2:** Secondary outcomes.

Author et al. (Year)	Prenatal imaging findings (signs) and placental location	Delivery mode and gestational age (wks) of PAS cases	Criteria used to confirm the diagnosis of PAS at birth	PAS grades
Zelop et al. (1992)^19^	No prenatal imaging described as part of this study	All patients had CD and PH in the third trimester	Clinical: Abnormally adherent placenta HP: method not described	5 PC/4 PI/2 PP
Kupferminc et al. (1993)^23^	No prenatal imaging described as part of this study	All patients had CD and PH Mean GA 34.5 (SD 3.9) wks	Clinical: not described HP: method not described	not provided
Hung et al. (1999)^24^	No prenatal imaging described as part of this study	18 patients had a vaginal birth and 10 had CD (including 2 PH) Mean GA 38.1 (SD 2.2) wks	Clinical: Difficult manual, piecemeal removal of placenta; sonographic evidence of retained placental fragments requiring curettage after vaginal delivery; heavy bleeding from implantation site after placental removal during cesarean managed conservatively with excision of part of the uterine wall and the attached placenta, or sewing the bleeding defects HP: method not described	not provided
Butler et al. (2001)^25^	US: no description of ultrasound signs used to evaluate PAS or confirm placental location	All PAS cases had CD and PH Mean GA at delivery not reported	Clinical: Abnormally adherent placenta HP: method not described	not provided
Wehrum et al. (2011)^26^	US: PAS sign (lacunae). TVS was used to confirm the placental location	All 45 cases had CD and all 13 PAS had PH Median GA 35 [IQR 34;36] wks	Clinical: not described HP: method not described	5 PC/6 PI/2 PP
Dreux et al. (2012)^27^	US: no description of ultrasound signs used to evaluate PAS or confirm placental location	34 patients had a conservative management, and 35 had a surgical management. Mode of delivery and GA at delivery not reported	Clinical: Absence of cleavage plane and with failure of gentle attempts to remove the placenta and or evidence of gross placental invasion HP: method not described	not provided
Desai et al. (2014)^28^	US: no description of ultrasound signs used to evaluate PAS or confirm placental location	All patients with PAS had CD and PH GA at delivery not reported	Clinical: not described HP: method not described	Not provided
Duzyj et al. (2015)^29^	US: no description of ultrasound signs used to evaluate PAS or confirm placental location	21 patients with PAS CD and PH; 1 had CD and PMR and 1 had vaginal birth with 2nd HT Median GA 34 [IQR 30–36] wks	Clinical: not described HP: method not described	5 PC/11 PI/7 PP
Thompson et al. (2015)^30^	US and MRI: no description of ultrasound signs used to evaluate PAS or confirm placental location	All patients with PAS had CD and PH GA range (*n* = 17) 29–39 wks	Clinical: not described HP: method not described	6 PC/ 3 PI/ 8 PP
Biberoglu et al. (2016)^31^	US: PAS signs including MT, absence of clear space, increased retroplacental vascularity and placental bulging. TVS was used to confirm the placental location	24 patients with PAS had CD and PH and 9 had CD with conservative management Mean GA (*n* = 33) 35.7 (SD 2.4) wks	Clinical: Abnormal placental attachment HP: method not described	17 PC&PI/ 18 PP
Ersoy et al. (2016)^32^	US: no description of ultrasound signs used to evaluate PAS or confirm placental location	All patients with PAS had CD and PH GA at delivery not reported	Clinical: not described HP: absence of decidua with direct attachment of villi to myometrium^53^	Not provided
Oztas et al. (2016)^33^	US: no description of ultrasound signs used to evaluate PAS or confirm placental location	27 patients had a CD. Management not described GA at delivery not reported	Clinical: not described HP: absence of decidua with direct attachment of villi to myometrium^53^	Not provided
Oztas et al. (2016)^34^	US: no description of ultrasound signs used to evaluate PAS or confirm placental location	61 patients had CD with conservative management (Mean (SD) GA 34.4 (3.7)) and 51 had CD and PH (Mean (SD) GA (CD&PH): 34.0 (4.0))	Clinical: not described HP: method not described	Not provided
Einerson et al. (2017)^35^	No prenatal imaging reported as part of this study	All patients with PAS had CD and PH GA at delivery not reported	Clinical: not described HP: disruption of the decidua and attachment or invasion of the trophoblast into the myometrium	Not provided
Buke et (2018)^36^	No prenatal imaging reported as part of this study	Mode of delivery and management not described Mean (SD) GA at delivery: 34.9 (0.7)	Clinical: not described HP: method not described	Not provided
Uyanikoglu et al. (2018)^37^	US: PAS signs including MT and lacunae	Mode of delivery and management not described Mean (SD) GA at delivery: 35.0 (2.1)	Clinical: not described HP: method not described	All cases described as PP
Uyanikoglu et al. (2018)^38^	US: PAS signs including MT, absence of clear space, increased retroplacental vascularity and bridging vessels	8 patients had CD and PH, 14 had CD, PMR and B‐Lynch suture Mean (SD) GA at delivery: 34.6 (2.7)	Clinical: not described HP: extreme trophoblastic invasion that involved the serosa of the uterus	All cases described as PP
Berezowsky et al. (2019)^39^	No prenatal imaging specified as part of this study	10 patients had CD and PH Median (IQR) GA at delivery: 38.0 (36, 39)	Clinical: partial or total failure of manual removal of the placenta, failure of gentle attempts to remove it during the third stage of labor and/or evidence of gross placental invasion at the time of CD HP: not described	Not provided
Penzhoyan et al. (2019)^40^	US and MRI: no description of ultrasound signs used to evaluate PAS or confirm placental location	Three patients had CD and PH Mean GA at delivery not provided	Clinical: not described HP: method not described	22 PC/ 2 PI/ 1 PP
Al Khan et al. (2020)^41^	US and MRI: no description of ultrasound signs used to evaluate PAS or confirm placental location	All patients had CD and PH Mean (SD) GA ranging 33.5 (0.2) for previa PAS to 34.2 (0.03) for PAS non‐previa	Clinical: not described HP: method not described	7 PC/ 49 PI& PP
Shainker et al. (2020)^42^	No prenatal imaging specified as part of this study	All patients had CD and PH Median (IQR) GA at delivery: 35.1 (0.8)	Clinical: placenta adherent to the uterine wall without easy separation from the placental bed HP: method not described	Not provided
Wang et al. (2020)^43^	US: no description of ultrasound signs used to evaluate PAS or confirm placental location	Mode of delivery and management not described Mean (SD) GA at delivery: 38.8 ± 1.3	Clinical: not described HP: method not described	Not provided
Faraji et al. (2021)^44^	US and MRI: no description of ultrasound signs used to evaluate PAS or confirm placental location	All patients but 1 had CD. Management not described GA at delivery not reported	Clinical: not described HP: absence of decidua between the placental villi and myometrium^53^ with various degrees of villous invasion	29 PC/ 8 PI/ 8 PP
Ozler et al. (2021)^45^	US: PAS signs including the loss of a clear zone and placental lacunae	Mode of delivery and management not described Median (IQR) GA at delivery: 36.6 (1.6)	Clinical: abnormal adherence of placenta HP: Villi invading all layers of the underlying uterine wall	Not provided
Sahin et al. (2021)^46^	US: PAS signs including loss of clear zone, bladder wall disruption, lacunae, bridging vessels and increased retroplacental vascularization. TVS used in all cases	22 patients had CD and PH, 10 had CD and PMR with Bakri balloon, 10 CD and PMR with B‐Lynch compression suture Mean (SD) GA at delivery: 34.5 (1.6)	Clinical: Absence of a plane of cleavage between the placenta and decidua myometrium, difficulty in full or partial placental detachment from the uterus, or visible invasion of the bladder HP: Absence of decidua^53^ or the presence of smooth muscle fibers in contact with placental villi	18 PC/ 16 PI/ 8 PP
Schwickert et al. (2021)^47^	US: PAS signs including loss of clear zone, bladder wall disruption, lacunae, bridging vessels, and increased retroplacental vascularisation	44 patients had CD and PH, 7 had CD and PMR, 4 had CD and manual removal, and 4 had CD with placental left in situ Median (IQR) GA at delivery: 35.0 (1.0)	Clinical: FIGO grades.^54^ HP: not described	6 PC/ 6 PI/ 32 PP
Wang et al. (2021)^48^	US: no description of ultrasound signs used to evaluate PAS or confirm placental location	Mode of delivery and management not described Mean (SD) GA at delivery: 38.9 (1.3)	Clinical: not described HP: method not described	Not provided
Wang et al. (2021)^49^	US and MRI: no description of ultrasound signs used to evaluate PAS or confirm placental location	Mode of delivery and management not described Mean GA at delivery not reported	Clinical: not described HP: method not described	Not provided
Ozler et al. (2022)^50^	US: standardized PAS ultrasound signs.^52^	Mode of delivery and management not described Mean (SD) GA at delivery: 36.6 (1.7)	Clinical: FIGO grades^54^ HP: method not described	12 PC/ 6 PI/ 12 PP

Abbreviations: CD, cesarean delivery; FIGO, Federation of Gynecology and Obstetrics; GA, gestational age; HP, histopathology; IQR, interquartile range; MRI, magnetic resonance imaging; MT, myometrial thinning; PC, placenta creta; PI, placenta increta; PP, placenta percreta; PH, peripartum hysterectomy (primary); PI, Placenta increta; PP, Placenta percreta; PMR, Partial myometrial resection; 2nd PH, secondary PH; SD, standard deviation; TVS, transvaginal ultrasound; US, ultrasound.

Out of 20 studies that reported on the management of patients with PAS, 19 described CD with peripartum hysterectomy (PH) as the main management strategy (Table 2).^23^, ^24^, ^25^, ^26^, ^28^, ^29^, ^30^, ^31^, ^32^, ^34^, ^35^, ^38^, ^39^, ^40^, ^41^, ^42^, ^46^, ^47^, ^51^ The gestational age at delivery ranged between 34 and 39 weeks. The clinical and histopathologic criteria used to confirm the diagnosis of PAS at birth were described in 11 and seven studies, respectively. Four authors^32^, ^33^, ^44^, ^46^ used the histologic criteria described by Irving and Hertig in 1937,^53^ two authors^47^, ^50^ used the 2019 International Federation of Gynaecology and Obstetrics (FIGO) classification for the clinical diagnosis of PAS,^54^ and the remaining studies did not report on the histopathologic criteria. The different grades of accreta placentation in the cases confirmed as PAS at birth were reported in 11 studies.^19^, ^26^, ^29^, ^30^, ^31^, ^40^, ^41^, ^44^, ^46^, ^47^, ^50^

### 3.2 Association between common maternal biomarkers and PAS

Higher level of AFP (odds ratio (OR) 9.73, 95% CI 2.25, 42.10; four studies^19^, ^23^, ^24^, ^25^; *I* ^2^ = 57%) and free β‐hCG (OR 4.06, 95% CI 1.72, 9.57; single study)^24^ were associated with PAS at birth (Figure S1). Patients with PAS at birth also had higher levels of PAPP‐A in pregnancy (Mean difference (MD) 0.52, 95% CI 0.36, 0.68; single study)^40^ but lower levels of PlGF (MD −36.14, 95% CI −45.18, −25.09; two studies^31^, ^38^; *I* ^2^ = 49%) compared with normal controls. The association between VEGF levels and PAS at birth was inconclusive (Standardized MD −1.21, 95% CI −2.48, 0.06; three studies^31^, ^38^, ^47^; *I* ^2^ = 94%).

## 4 DISCUSSION

Our scoping review has identified 6 cohorts and 23 case–control studies that have evaluated 34 different protein biomarkers in the serum of patients with PAS, clinically or pathologically confirmed at birth. Based on these papers, we found that accreta placentation has been reported to be associated with changes in the levels of established maternal serum biomarkers including biomarkers previously used for the screening fetal neural tube defects (AFP) and aneuploidy (free β‐hCG) (Figure S1) and new biomarkers used more recently for the screening preeclampsia and fetal growth restriction (PlGF, PAPP‐A) (Table 1; Table S1). In addition, we found that 30 other protein biomarkers have been tested in the antenatal evaluation of PAS at different gestational age including at delivery and for which usefulness after the mid‐pregnancy detailed fetal anatomy scan is likely to be limited for the screening of patients at risk of PAS at birth. Overall, we identified major evidence gaps in the literature on the clinical protocols used to identify and report on patients with a high probability of PAS at birth. In particular, important key items are lacking such as unavailability of suitable cutoffs for most biomarkers, variability of gestational age at sampling and the potential overlap of the marker values with other placental‐related complications of pregnancy.

Maternal serum biomarkers previously and currently used for the screening of pregnancy complications are available in most routine laboratory in high‐income countries and many middle‐income countries. Measurements of AFP in amniotic fluid and MS were first introduced in the 1970s as an aid to the diagnosis and of neural tube defects.^55^, ^56^, ^57^ All seven studies (Table 1; Table S1) reporting on MSAFP included in the present review found a higher level of AFP in the second trimester serum samples of patients with PAS confirmed at birth compared with controls (OR 9.72; 95% CI 2.25, 41.99). High MSAFP levels have been associated with other placenta anomalies such intervillous thrombosis and infarcts^58^, ^59^ suggesting that a breakdown in the placental barrier is the main factor of high MSAFP in patients with pregnancies complicated by PAS.

Intact hCG was first used in combination with AFP and estriol in the second trimester of pregnancy^60^ and then replaced with MS free β‐hCG and PAPP‐A in first trimester screening protocols.^61^ We identify 10 studies reporting on the levels of hCG molecules in patients with PAS at birth. The largest study^24^ was a cohort of 9349 births which found a 25% incidence of PAS in patients with free β‐hCG >2.5 MoM in the second trimester. High MS first trimester free β‐hCG^36^ and second trimester MS intact hCG^27^, ^39^ and lower hCG‐H^35^ were also reported in PAS compared with normal controls. One study reported a lower MS first trimester‐free β‐hCG^30^ and the remaining four studies^28^, ^34^, ^40^, ^41^ found no difference in hCG levels between PAS cases and controls. Oxidizing conditions promote combination of the subunits in vitro and thus the physiologic oxidative stress at the end of the first trimesters^62^ and temporal relationship with the hCG concentration peak^63^ may influence the pattern of secretion in vivo and explain the variation in results between studies.

Only one previous study reviewed the association between third‐trimester MS levels of PIGF, VEGF and Soluble fms‐like tyrosine kinase (sFLT‐1) and PAS.^64^ This review included studies with no confirmation of PAS at birth and found lower sFlt‐1 levels in PAS cases compared to controls but no significant difference in the VEGF and PIGF MS levels. Lower first trimester MSPIGF levels have been found in patients who developed preeclampsia later in pregnancy^65^ and sFLT‐1/PIGF ratio is used to rule out the development of preeclampsia with 4 weeks after the test.^66^ In the present review, we identified six studies^26^, ^31^, ^38^, ^44^, ^47^, ^49^ reporting on levels of VEGF, PlGF, and/or sFLT‐1 in the third trimester, one on MS PlGF alone in the first trimester (Table 1; Table S2).^43^ Lower MSVEGF levels were reported by most authors in patients with PAS^26^, ^38^, ^47^ whereas PlGF levels were lower in one study^31^, ^38^ and higher in three.^26^, ^43^, ^44^

Low MS PAPP‐A are associated with fetal aneuploidy^61^ and adverse perinatal outcomes including intrauterine growth restriction, miscarriage and stillbirth.^65^, ^66^, ^67^, ^68^ In the present review, six studies reported on first trimester MS PAPP‐A in patients with PAS at birth.^28^, ^30^, ^34^, ^36^, ^40^, ^48^ All except one^40^ reported a higher level in PAS cases than in controls.

Our scoping review has both strengths and limitations. We performed comprehensive literature searches for all know common protein biomarkers, making it unlikely that we missed any significant publications and all the studies included in our systematic review reported that the diagnosis of PAS was confirmed at birth, clinically during delivery and/or by histopathologic examination. The main limitation of our study was the high heterogeneity between the study design restricting the possible use of a meta‐analysis. One of the many challenges in the evaluation of the data from pregnancies complicated by PAS is assessing the accuracy of the diagnosis of PAS at birth. Overall, only 11 of the 29 studies included in our systematic review provided data on the severity of PAS as evaluated by the grades of accreta placentation. The high proportion of placenta percreta in some studies^31^, ^36^, ^38^, ^40^, ^47^ which refers to uterine remodeling rather than accreta placentation,^69^, ^70^ suggest that in those series the diagnosis of PAS at birth may have been overestimated. Another possible confounding factor is the selection by a few authors of a study group of patients with placenta previa accreta that presented with vaginal bleeding in the third trimester.^26^, ^31^ Although there is no evidence that previa placentation with or without PAS is associated with abnormal feto‐placental development,^71^ chronic bleeding may have an impact on biomarker measurements, in particular when performed on admission to hospital and/or immediately before delivery.^32^, ^33^, ^37^, ^38^, ^47^

Placental lobules developing within the scar area often receive their blood supply directly from the radial or arcuate arteries and the corresponding EVTs migrate directly next to these vessels. The entry of high‐velocity blood flow inside the intervillous leads to the formation of the lacunae^72^ but also probably a focal oxidative stress^73^ and sheer stress^74^ which could explain the changes in the MS level of proteins synthetized by the villous syncytiotrophoblast, even in the absence of microscopic villous damage.^10^ Oxidative and sheer stresses are probably also part of the mechanisms that leads to the progressive deposition of fibrinoid deposition in the accreta areas at the utero‐placental interface.^10^ Thus, it is essential that biomarkers are evaluated at different gestational ages and that further studies include a detailed description of the intra‐operative gross findings and in particular the size of the accreta villous tissue and location within the scar area.

## 5 CONCLUSION

Although ultrasound imaging has a high accuracy rate in identifying patients with a high probability of PAS at birth when perform by an expert operator, the corresponding expertise is not widely available and thus missed diagnosis is common. The usefulness of maternal serum biomarkers used in the screening and diagnosis of PAS is currently difficult to evaluate due to evidence gaps in our knowledge regarding limited data on the change in their levels in PAS with advancing gestational age, the mechanisms leading to these changes and the lack of correlation between biomarker levels and changes of the utero‐placental anatomy on prenatal imaging at the time of testing and gross features of the LUS at delivery. By contrast, the bioassays used in the screening of fetal aneuploidy, fetal neural tube defects, fetal growth restriction, and preeclampsia are widely available including in middle‐income countries. Multicentric prospective studies examining these biomarkers in individuals with clinically and histopathologically confirmed PAS at birth and with carefully selected, gestational age‐matched controls are needed.

## AUTHOR CONTRIBUTIONS

All authors contributed to the initial literature search. The systematic review was performed by Matthew Givens and Ivaila Valcheva. The data collection was performed by Matthew Givens, Ivaila Valcheva, and Eric Jauniaux, and the data analysis was performed by Ewelina Rogozińska. All authors contributed to the writing of the manuscript.

## CONFLICT OF INTEREST STATEMENT

The authors report no conflict of interest.

## Supporting information


Figure S1.

Table S1.
