# Supplementary figures and images for: Evaluation of maternal serum protein biomarkers in the prenatal evaluation of placenta accreta spectrum: A systematic scoping review
Source: Acta Obstet Gynecol Scand. 2024 Jul 14;103(12):2335–47. doi: 10.1111/aogs.14918 (PMC11610010; doi:10.1111/aogs.14918)

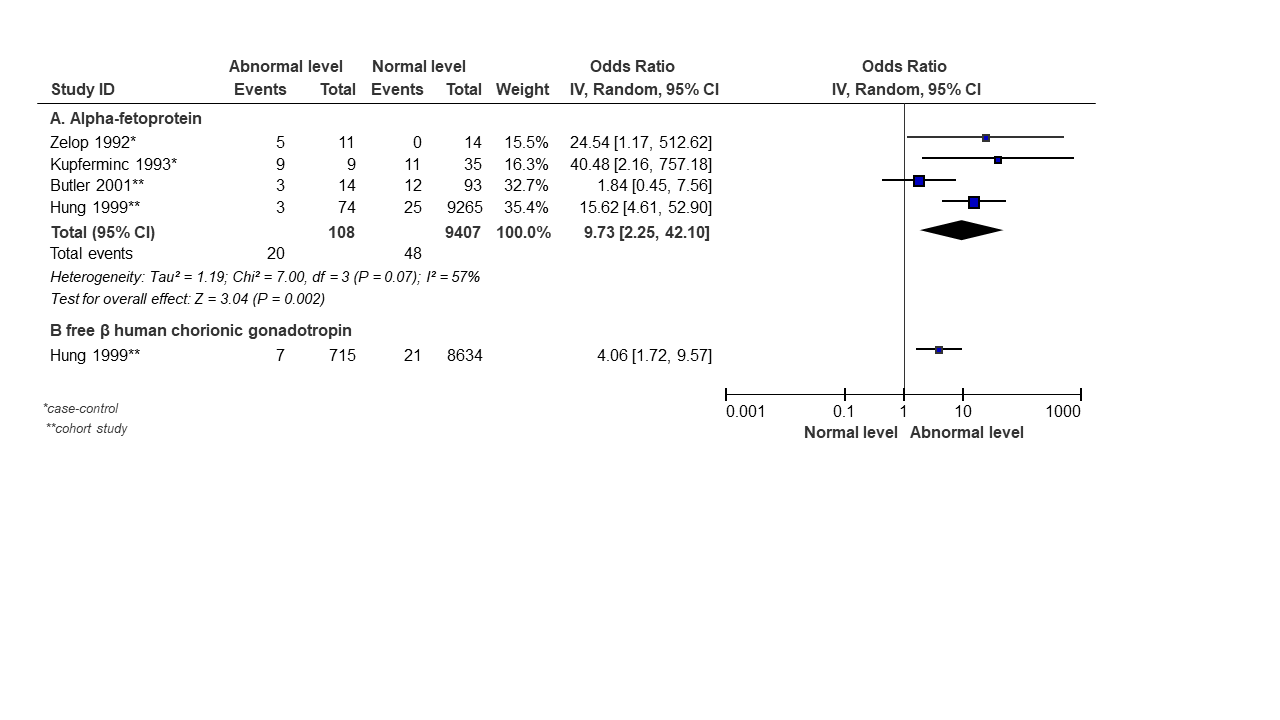

Supplement: Supplementary file 1 — Figure S1. [file AOGS-103-2335-s001.tif]
