# Supplementary material for: Evaluation of maternal serum protein biomarkers in the prenatal evaluation of placenta accreta spectrum: A systematic scoping review
Source: Acta Obstet Gynecol Scand. 2024 Jul 14;103(12):2335–47. doi: 10.1111/aogs.14918 (PMC11610010; doi:10.1111/aogs.14918)
Supplement: Supplementary file 2 — Table S1. [file AOGS-103-2335-s002.docx]

**Table S1:** Overview of extracted data on biomarkers results (significant difference are highlighted in bold)

| **Author et al**  **(Year)** | **MS biomarker & cut-off**  **(GA)** | **Results** |
| --- | --- | --- |
| Zelop et al (1992)^19^ | AFP >2 MoM (2^nd^ Trimester) | 5/11 (45.5%) PAS & 0/14 (0%) previa non-PAS |
| Kupferminc et al (1993)^24^ | AFP > 2.5 MoM (2^nd^ Trimester) | 9/20 (45.0%) PAS & 0/24 (0%) controls (including 12 previa non-PAS) |
| Hung et al (1999)^25^ | AFP >2.5 MoM  Free β-hCG >2.5 MoM  (14-22 wks) | AFP: 3/28 (10.7%) PAS & 71/9321 (0.76%) controls (including 68 previa non-PAS)  Free β-hCG: 7/28 (25.0%) PAS & 708/9321 (7.60%) controls (including 68 previa non-PAS) |
| Butler et al (2001)^26^ | AFP > 2.0 MoM (15-20 wks) | 3/15 PAS (20.0%) & 14/92 (15.2%) previa non-PAS |
| Wehrum et al (2011)^27^ | No cut-off specified for:  VEGF (pg/mL)  PlGF (pg/mL)  sFlt (pg/mL)  (28-34 wks) | Median [IQR] VEGF (pg/mL): PAS= 0.8 (0.02;3.4) vs previa non-PAS= 6.5 [2.7;10.5]; (**P=0.02**)  Median [IQR] PlGF (pg/mL): PAS= 823 [292;1,029] vs previa non-PAS= 370 [249;572]; (P=0.137)  Median [IQR] sFlt (pg/mL): PAS= 1,343 [792;2,325] vs previa non-PAS= 1,301 [821;1,661]; (P=0.758) |
| Dreux et al (2012)^28^ | AFP (MoM)  Intact hCG (MoM)  (2^nd^ Trimester) | Absolute number above and below the cut-offs not reported  AFP>2.5 MoM OR for PAS 9.7  hCG> 2.5 MoM OR for PAS 8  Combined MSAFP and hCG >2.5 MoM OR for PAS 32.2 |
| Desai et al (2014)^29^ | No cut-off specified for:  PAPP-A (MoM)  Free β-hCG (MoM)  (1^st^ Trimester) | Median [IQR] PAPP-A (MoM): PAS= 1.68 [0.5;3.26] vs non-PAS previa = 0.98 [IQR not provided]; (**P=0.002**)  Median [IQR] Free β-hCG (MoM): PAS= 1.00 [0.33;1.63] vs non-PAS previa= 1.01 [IQR not provided]; (P=0.416) |
| Duzyj et al (2015)^30^ | No cut-off specified for:  Soluble E-CAD (pg/mL)  (25-35 wks) | Mean [SD] soluble E-CAD (pg/mL): PAS= 59.2 [26.7] vs previa non-PAS = 52.3 [41.1] (P= 0.657) |
| Thompson et al (2015)^31^ | No cut-off specified for:  PAPP-A (MoM)  Free β-hCG (MoM)  (1^st^ Trimester) | Median PAPP-A (MoM): PA= 1.22 vs previa non-PAS= 1.05; (P= 0.16)  Median free β -hCG (MoM): PAS= 0.81 vs previa non-PAS= 1.08; (P=0.06) |
| Biberoglu et al (2016)^32^ | No cut-off specified for:  VEGF (pg/mL)  PlGF (pg/mL)  sFlt (ng/mL)  (3^rd^ Trimester) | Mean [SD] VEGF (pg/mL): PAS-accreta/increta= 4.885 [1.480] vs PAS-percreta= 6.34 [2.341] vs previa non-PAS= 6.405 [2.819] vs normal control= 5.454 [2.541]; (P=0.131)  Mean [SD] PlGF (pg/mL): PAS-accreta/increta= 77.802 [37.101] vs PAS-percreta= 93.986 [49.098] vs previa non-PAS= 95.307 (36.153) vs normal control= 81.498 [29.522]; (P=0.276)  Median [IQR] sFlt-1 (ng/mL): PAS-accreta/increta= 1.427 [1.315;1.689] vs PAS-percreta= 1.403 [1.299;1.607] vs. previa non-PAS= 1.422 [1.332;1.707] vs normal control= 1.387 [1.312;1.666]; (P=0.196) |
| Ersoy et al (2016)^33^ | Troponin I >0.0045 ng/mL  ProBNP >125.85 pg/mL  No cut-off specified for:  CK (IU/L)  CK-MB (ng/mL)  (at delivery) | Mean [SD] Troponin I (ng/mL): PAS= 0.054 [0.008] vs previa non-PAS= 0.52 [0.008]; (P=NS) & vs normal control= 0.003 [0.0009]; (P=**0.015**)  Mean [SD] ProBNP (pg/mL): PAS= 143.22 [76.32] vs previa non-PAS= 62.85 [50.95]; (**P<0.001**) & vs normal control= 46.11 [25.35]; (p<0.001)  Mean [SD] CK (IU/L): PAS= 76.16 [57.41] vs previa non-PAS= 59.94 [65.14]; (P=NS) & vs normal control= 82.36 [51.71]; (P=NS)  Mean [SD] CK-MB (ng/mL): PAS= 19.25 [8.23] vs previa non-PAS= 20.65 [1.861]; (P=NS) & vs normal control= 25.56 [11.74]; (P=NS) |
| Oztas et al (2016)^34^ | No cut-off specified for:  TRAIL-R2 (pg/mg)  (at delivery) | Mean [SD] TRAIL-R2 (pg/mg): PAS= 19.85 [5.72] vs previa non-PAS = 25.99 [6.98]; (**P=0.006**) & vs normal control= 25.87 [8.08]; (**P=0.005**) |
| Oztas et al (2016)^35^ | AFP>1.25 MoM  Cut-offs not provided for:  PAPP-A (MoM)  Free β-hCG (MoM; 1st trimester)  uE3 (MoM)  Free β-hCG (MoM; 2nd trimester) | Mean [SD] AFP (MoM) PAS-conservative management= 1.18 [0.16] vs PAS-hysterectomy= 1.28 [1.065] vs previa non-PAS= 0.87 [0.37]; (**P<0.001**)  Mean [SD] PAPP-A (MoM) PAS-conservative management= 1.19 [0.80] vs PAS-hysterectomy= 1.75 [1.41] vs previa non-PAS= 1.01 [0.96]; (P=NS)  Mean [SD] free β-hCG (MoM) PAS PAS-conservative management= 1.11 [0.62] vs PAS PAS-hysterectomy= 1.46 [1.02] vs previa non-PAS= 1.21 [0.82]; (P=NS)  Mean [SD] uE3 (MoM) PAS PAS-conservative management= 0.96 [0.50] vs PAS PAS-hysterectomy= 1.10 [0.49] vs. previa non-PAS= 0.88 [0.34]; (P=NS)  Mean [SD] free β-hCG (MoM) PAS PAS-conservative management= 1.39 [0.85] vs PAS-hysterectomy= 1.19 [0.56] vs previa non-PAS= 1.13 [0.48]; (P=NS) |
| Einerson et al (2017)^36^ | hCG-H ≤7.6 μg/L (2^nd^ and 3^rd^ trimester) | Mean [SD] hCG-H (µg/L) PAS= 7.8 [5.9] vs controls b= 11.8 [8.8]; (**P=0.03**) |
| Buke et (2018)^37^ | PAPP-A >1.0 MoM  Free β-hCG >1.0 MoM  (1^st^ trimester) | PAPP-A: 13/19 (68.4%) PAS & 32/69 (46.4%) previa non-PAS control  Median [range] PAPP-A (mIU/mL) PAS: 1.2 [0.4–3.1] vs previa non-PAS: 0.86 [0.25–1.8]; (**P=0.045**)  Free β-HCG: 9/19 (47.4%) PAS; Controls not reported  Median [range] free β -hCG (mIU/mL) PAS: 1.4 [0.3–4] vs previa non-PAS: 0.93 [0.3–1.8]; (**P=0.042**) |
| Uyanikoglu et al (2018)^38^ | Cut-offs not provided for:  Native thiol (µmol/L)  Total thiol (µmol/L)  Disulfide (µmol/L)  IMA (IU/mL)  (at delivery) | Mean [SD] native thiol (µmol/L): PAS= 216.95 [37.52] vs controls^b^= 234.57 [34.46]; (**P=0.034**)  Mean [SD] total thiol (µmol/L): PAS= 255.57 [38.18] vs controls^b^= 276.84 [41.32]; (**P=0.021**)  Mean [SD] disulfide (µmol/L): PAS= 20.18 [6.84] vs controls^b^= 21.28 [8.3]; (**P=0.051**)  Mean [SD] IMA (IU/mL): PAS= 0.83 [0.09] vs controls^b^= 0.77 [0.08]; (**P=0.006**) |
| Uyanikoglu et al (2018)^39^ | Cut-offs not provided for:  VEGF (pg/mL)  PlGF (pg/mL)  sFlt-1 (ng/mL)  (at delivery) | Mean [SD] VEGF (pg/mL): PAS= 39.18 [11.98] vs control^b^= 85.87 [18.05]; (**P<0.001**)  Mean [SD] PlGF (pg/mL): PAS= 39.94 [12.00] vs control^b^= 76.65 [21.49]; (**P<0.001**)  Mean [SD] sFLt-1 (ng/mL): PAS: 236.28 [48.44] vs control^b^= 276.30 [77.72]; (**P<0.05**) |
| Berezowsky et al (2019)^40^ | Cut-offs for >75th percentile  AFP >1.46 MoM  hCG >1.71 MoM  No cut-offs provided for: uE3 (MoM)  (2^nd^ trimester) | Median [IQR] AFP (MoM): PAS non previa= 1.19 [1.01;1.4] vs previa non-PAS: 1.13 [0.91;1.41) vs previa PAS= 1.15 [1.0;1.45) vs normal control= 1.08 (0.82;1.32); (**P=0.027**)  Median [IQR] hCG (MoM): PAS non previa= 1.59 [1.2;2.19] vs previa non-PAS= 1.12 [0.81;1.61) vs previa PAS= 1.39 [0.9;1.7] vs normal control= 1.02 [0.69;1.46]; (**P<0.001**)  Median [IQR] UE3 (MoM): PAS non previa= 0.99 [0.8;1.18] vs previa non-PAS= 0.98 [0.85;1.14] vs previa PAS= 0.90 [0.76;1.06] vs control= 0.98 [0.78;1.22]; (P=0.776) |
| Penzhoyan et al (2019)^41^ | No cut-offs provided for:  PAPP-A (MoM)  Free β-hCG (MoM)  (1^st^ trimester) | Mean [SD] PAPP-A (MoM): PAS= 1.30 [0.85] vs previa non-PAS= 1.91 [1.52]; (P=0.640) & vs control= 1.07 [0.47]; (P=0.311)  Mean [SD] free β -hCG (MoM): PAS= 1.16 [0.65] vs previa non-PAS= 1.46 [0.19]; (P=0.646) & vs control= 1.08 [0.69]; (P=0.750) |
| Al Khan et al (2020)^42^ | No cut-offs provided for:  hCG-H (pmol/L)  Decorin (pg/mL)  IL-8 (pg/mL)  (> 25 weeks) | Mean [SD] hCG-H (pmol/L): increta/percreta= 107 [16] vs increta/percreta-previa= 104 [2] vs accreta-previa= 107 [6] vs previa= 112 [9] vs normal controls= 128 [11]; (P=NS)  Mean [SD] decorin (pg/mL): increta/percreta= 38 [6] vs increta/percreta-previa= 35 [2] vs previa= 57 [9] vs normal controls= 36 [2]; (P=NS)  Mean [SD] IL-8 (pg/mL): PAS-previa = 33.2 [2.7] vs previa non-PAS= 14.7 [4.1]; (**P<0.01**) |
| Shainker et al (2020)^43^ | No cut-offs provided for:  Antithrombin III (mg/mL)  PAI-1 (ng/mL)  sTie2 (ng/mL)  sVEGFR-2 (ng/mL)  (3^rd^ trimester) | Median [IQR] Antithrombin III (mg/mL): PAS= 240.4 [177.9;253.8] vs controls^c^= 150.3 [130.2;174.5]; (**P=0.002**)  Median [IQR] PAI-1 (ng/mL): PAS= 4.1 [3.7;5.4 vs controls^c^= 7.1 [5.8;10.3]; (**P<0.001**)  Median [IQR] Soluble Tie2 (ng/mL): PAS= 9.0 [7.5;9.8] vs controls^c^= 5.9 [5.5;6.8]; (**P=0.02**)  Median [IQR] sVEGFR-2 (ng/mL): PAS= 13.5 [11.6;16.8] vs controls^c^= 10.4 [9.3;12.9]; (**P=0.003**) |
| Wang et al (2020)^44^ | No cut-offs provided for:  PlGF (MoM)  (1^st^ trimester) | Mean [SD] PlGF (MoM): PAS= 1.27 [0.43] vs previa non-PAS= 1.00 [0.44]; (**P=0.03**) & vs normal control= 0.96 [0.42]; (**P<0.001**) |
| Faraji et al (2021)^45^ | PlGF >63.55 pg/mL  No cut-offs provided for:  VEGF (pg/mL)  (3rd trimester) | Median [IQR] PLGF (pg/mL): PAS= 88.4 [209.5] vs control^b^= 35.9 [51]; (**P<0.001**)  Median [IQR] VEGF (pg/mL): PAS= 229.8 [265.95) vs control^b^= 285.9 [295.1]; (P=0.17) |
| Ozler et al (2021)^46^ | IL-33 >47.26 pg/mL  No cut-offs provided for:  IL-6 (pg/mL)  CRP (mg/L):  (> 26 weks) | Mean [SD] IL-33 (pg/mL): PAS= 60.97 [5.82] vs previa non-PAS= 46.04 [9.54]; (**P=0.011**) & vs normal control= 45.99 [9.91]; (**P=0.011**)  Mean [SD] IL-6 (pg/mL): PAS= 17.51 [5.24] vs previa non-PAS= 15.70 [1.78]; (P= NS) & vs normal controls= 11.36 [4.84]; (**P=0.045**)  Mean [SD] CRP (mg/L): PAS= 0.96 [0.17] vs previa non-PAS= 0.99 [0.22]; (P= NS) & vs normal controls= 0.91[0.06]; (P= NS) |
| Sahin et al (2021)^47^ | PP-13 >312.58 pg/mL  (3rd trimester) | Mean (SD) PP-13 (pg/mL): PAS= 650.32 [387.33] vs control^b^= 231.43 [94.33]; (**P<0.001**) |
| Schwickert et al (2021)^48^ | VEGF >328.0 pg/mL  NT-proBNP >303.5 pg/mL  (at delivery) | Median [IQR] VEGF (pg/mL): PAS= 285 [248;322] vs controls^d^= 391 [356;426]; (**P=<0.01**)  Median [IQR] NT-proBNP (pg/mL): PAS= 329 [287;385] vs controls^d^= 295 [273;356]; (**P=0.03**) |
| Wang et al (2021)^49^ | No cut-offs provided for:  PAPP-A (MoM)  (1^st^ trimester) | Median [range] PAPP-A (MoM): PAS 1.39 [0.17;4.78] vs previa non-PAS: 0.85 (0.34;2.81); (P=0.009) & vs normal control 0.98 (0.28;2.48); (**P<0.001**) |
| Wang et al (2021)^50^ | VEGF < 87.37 ng/mL  sFLT-1 > 1.558 ng/mL | Mean/median values are not available |
| Ozler al (2022)^51^ | TSH< 2.16 mIU/L  TgAB< 2.70 IU/mL  No cut-offs provided for:  T3 (ng/dL)  T4 (ng/dL)  TPOAb (ng/dL)  (3^rd^ trimester) | Mean [SD] TSH (mIU/L): PAS= 1.21 [0.96] vs previa non-PAS= 2.59 [1.53] vs normal controls= 3.50 [2.33]; (**P=<0.001**)  Mean [SD] TgAb (IU/mL): PAS= 2.17 [1.57] v. previa non-PAS= 4.47 [3.70] vs normal controls= 5.33 [3.34]; (**P <0.001**)  Mean [SD] T3 (ng/dL): PAS= 0.156 [0.10] vs previa non-PAS= 0.160 [0.02] vs normal controls= 0.162 [0.04]; (P= NS)  Mean [SD] T4 (ng/dL): PAS= 0.134 [0.01] vs. previa, non-PAS= 0.126 [0.01] vs normal controls= 0.125 [0.06]; (P= NS)  Mean [SD] TPOAb (ng/dL): PAS= 0.140 [0.06] vs previa non-PAS= 0.023 [0.001] vs normal controls= 0.090 [0.004]; (P= NS) |

**Abbreviations (general):** dL= decilitre; IU= international units; IQR= interquartile range; MoM= multiple of the median; MS= maternal serum; mL= millilitre; ng= nanograms; NS= not significant; OR= odds ratios;; PAS= placenta accreta spectrum; pg= picograms; SD= standard deviation

**Abbreviations (biomarkers):** AFP= alpha-fetoprotein; ARES= anti-oxidant response elements; β-hCG= β subunit of human chorionic gonadotropin; CK= creatine kinase; CK-MB= cardiac form of CK; CRP= C-reactive protein; E-CAD= E-cadherin; hCG-H= hyperglycosylated human chorionic gonadotropin; IL= interleukin; IMA= ischemia modified albumin; NT-proBNP= N-terminal prohormone of brain natriuretic peptide; OSI= oxidative stress index; PAI-1= plasminogen activator inhibitor-1; PAPP-A= Pregnancy-associated plasma protein A; PlGF= placenta growth factor; PP-13= Placental Protein 13; ProBNP= Pro-brain natriuretic peptide; sFLT-1= Soluble fms-like tyrosine kinase; sVEGFR-2= Soluble VEGF receptor 2; T3= triiodothyronine; T4= thyroxine; TAS= total antioxidant status; TgAB= thyroglobulin antibodies; TOS= total oxygen species; TPOAb= Thyroid peroxidase antibodies; TRAIL-R2= TNF-related apoptosis-inducing ligand receptor-2; TSH= thyroid stimulating hormone; uE3= estriol; VEGF= Vascular endothelial growth factor.

^a^ p-values are multigroup comparisons

^b^ placental location in the control group is not specified

^c^ control group includes 4 with normal placentation, 5 with placenta previa, 1 with unknown placental location

^d^ control group includes 50 with normal placentation, 5 with placenta previa
